# The African strain of Zika virus causes more severe *in utero* infection than Asian strain in a porcine fetal transmission model

**DOI:** 10.1080/22221751.2019.1644967

**Published:** 2019-07-25

**Authors:** Daniel Udenze, Ivan Trus, Nathalie Berube, Volker Gerdts, Uladzimir Karniychuk

**Affiliations:** a Vaccine and Infectious Disease Organization-International Vaccine Centre (VIDO-InterVac), University of Saskatchewan, Saskatoon, Canada; b School of Public Health, University of Saskatchewan, Saskatoon, Canada; c Department of Veterinary Microbiology, Western College of Veterinary Medicine, University of Saskatchewan, Saskatoon, Canada

**Keywords:** Zika virus, *in utero* infection, fetus, pig, African strain, Asian strain, interferon alpha, cortisol

## Abstract

Studies in mice showed that African Zika virus (ZIKV) strains cause more damage in embryos. These studies, however, were limited to the mouse-adapted African MR766 strain or infection at early gestation. Here, we compared infection of Asian and African strains in the fetal pig model at midgestation. Both strains caused fetal infection. ZIKV was detected in placenta, amniotic membrane, amniotic fluid, fetal blood, and brain. The African strain produced more vigorous *in utero* infection as represented by more efficient virus transmission between siblings, and higher viral loads in fetal organs and membranes. Infection with both strains was associated with reduced fetal brain weight and increased number of placental CD163-positive cells, as well as elevated *in utero* interferon alpha and cortisol levels. This is the first large animal model study which demonstrated that African strain of ZIKV, with no passage history in experimental animals, can cause persistent infection in fetuses and fetal membranes at midgestation. Our studies also suggest that similar to Asian strains, ZIKV of African lineage might cause silent pathology which is difficult to identify in deceptively healthy fetuses. The findings emphasize the need for further studies to highlight the impact of ZIKV heterogeneity on infection outcomes during pregnancy.

## Introduction

Zika virus is a single-stranded RNA virus in the *Flaviviridae* family. Based on phylogenetic analysis, there are at least two distinct ZIKV lineages, specifically, the Asian lineage and African lineage. Asian strains are currently circulating in the western hemisphere and worldwide [1]. Zika virus of Asian lineage caused the 2015–2017 epidemic in the Americas [1] and the most recent outbreak in India [2]. It is well recognized that human infection with Asian ZIKV strains may result in severe fetal pathology and devastating congenital malformations in newborns and children [3]. Zika virus was first isolated in Uganda in 1947 [4]. Recent studies also showed that African strains are currently maintained in an enzootic cycle, including circulation in humans [5–10] and wild African primates [11]. However, in contrast to contemporary Asian strains, African strains have not been clearly linked with congenital infection in humans, and their epidemic potential is poorly understood. It has been suggested that ZIKV epidemic in the Americas and unusual for flaviviruses congenital pathology is a result, at least in part, of genetic mutations in the viral genome of Asian strains which affects mosquitoes’ vector competence [12] and mammalian host susceptibility [13]. Recent studies, however, demonstrated that African strains may cause more productive infection in mosquitoes and more severe pathology in mouse embryos [14–17] Thus, there are concerns that underdeveloped medical surveillance overlooked ZIKV infection and associated congenital pathology in Africa [18, 19]. Moreover, the majority of congenital infections in humans is subclinical [3] and is not associated with easily identifiable brain lesions or birth defects in newborns. Asymptomatic infection and potentially detrimental life-long health outcomes in deceptively healthy offspring evoke high concerns [3, 20, 21]; however, a majority of asymptomatic cases are likely not recorded in the Americas and probably in Africa [18].

Comparative *in vitro* studies demonstrated that ZIKV strains of both lineages could infect different cell lines and primary cells. However, outcomes of infection caused by African and Asian strains were cell-specific, which does not allow to predict infection phenotypes *in vivo* [22–25]

In non-human primate studies, rhesus macaques infected with the African MR766 strain showed viremia similar to animals infected with the Asian H/FP/2013 strain [26]. In two other studies, prolonged viremia was detected in macaques inoculated with the Asian PRVABC59 strain, but the African IbH_30656 strain induced low viremia or failed to establish infection [27, 28].

African strains showed more pathogenic phenotype in most studies in non-pregnant mice [29–32] One study, however, described similar infection patterns caused by a contemporary human clinical isolate and historical African strains [33]. In a comparative study, intrauterine inoculation in CD1 mice at 10 days of gestation with African (IbH_30656) and Asian (PRVABC59; FS13025; Pariaba) ZIKV strains resulted in similar maternal, uterine, placental, and fetal brain infection and comparable fetal death [34]. In another study, direct inoculation of embryos into the lateral brain ventricles with the African MR766 strain caused a more severe neuronal reduction, brain damage and postnatal mortality than inoculation with the Asian MEX1-44 strain [16]. In a most recent study [17], immunodeficient *Ifnar1^−/−^* mice crossed with wild-type sires and bearing heterozygous embryos with one intact *Ifnar1* haplotype, were inoculated subcutaneously at 7.5 days of gestation with the African (DAK-AR-41524) or Asian ZIKV (PRVABC59) strain. The vertical transmission mouse model showed that infection with the African strain leads to 100% fetal mortality, while the proportion of abnormal fetuses in mice inoculated with the Asian strain was 53.2% [17]. These pioneering mouse studies, however, were limited to the African MR766 strain [16], which was passaged numerous times in mice, or to inoculation at an embryo developmental period (7.5 and 10 days of gestation [17, 34]) corresponding to early human gestation.

In the present study, we used Asian and African ZIKV strains, which were not passaged in experimental animals, to compare infection phenotypes in the fetal pig model at midgestation. The fetal pig model recapitulates key aspects of human *in utero* ZIKV infection with virus persistence in the placenta, amniotic membrane, and fetal brain [35–37] The model is relevant because pigs are similar to humans in anatomy, physiology, immunology, and genetics [38]. Importantly for ZIKV research, pigs and humans have similar fetal brain development and postnatal brain growth [39]. Here, like in previous mouse [16, 34] and non-human primate ZIKV studies [40], we used *in utero* inoculation. Four fetuses (on average pigs have 12–16 fetuses) were inoculated with the Asian or African ZIKV strain at the middle stage of development and sampled 28 days later to compare *in utero* infection phenotypes.

## Materials and methods

### Experimental design

The low-passage, contemporary Asian ZIKV strain PRVABC59 [GenBank: KU501215.1] and historical African ZIKV strain DAK-AR-41524 (BEI resources) [Genbank: KY348860.1] were used in this study. Animal experiments were performed following the Canadian Council on Animal Care guidelines for humane animal use and were approved by the University of Saskatchewan's Animal Research Ethics Board. *In utero* inoculation and tissue sampling were performed as previously described [35, 36]. RNA extraction, RT-qPCR assays, infectious virus titration, serological, ZIKV-specific *in situ* hybridization (ISH), interferon alpha (IFN-α), and cortisol assays were performed as previously described [35, 36] (supplemental files).

### Statistical analysis

We used GraphPad PRISM7 software (GraphPad Software Inc., USA). Results were considered to be significantly different when *p* < 0.05. The number of dead fetuses and the number of fetuses with negative-strand ZIKV RNA in the cerebrum, placenta, amniotic membrane, and fetal blood plasma were compared with Yates-corrected χ^2^-test. Brain and body weights, *in utero* IFN-α and cortisol levels were compared with Kruskal–Wallis *H*-test. PCR data, infectious virus titers, and ZIKV-specific IgG Ab titers were compared with Mann–Whitney *U*-test. The number of CD163-positive cells in placental samples was compared with Kruskal–Wallis *H*-test. The relationship between distances from directly inoculated and trans-infected fetuses and ZIKV loads was accessed with linear regression analysis. Goodness-of-fit (r^2^) and statistical significance (p) were calculated for regression analysis.

## Results

### The African strain of ZIKV causes more vigorous trans-fetal infection than Asian strain

Four conceptuses (a fetus with fetal membranes) in each pig were inoculated with Asian and African ZIKV strains (two pregnant pigs per group) (Suppl. Figure S1(A-B), Table S1(A)). For inoculation, we used 10^6^ TCID_50_/fetus of the Asian PRVABC59 strain or the African DAK-AR-41524 strain. Inoculations were done at 50 days of gestation (gd) (the pregnancy in pigs is 114–115 days). Inoculation at 50 gd corresponds to infection in the middle/end of the second trimester in humans; during this period, like in the human fetal brain, the pig fetal brain undergoes a growth spurt [39]. Conceptuses from a control pig were exposed to virus-free media. Fetal tissues, membranes, and body fluids were sampled at 28 days after inoculation (Suppl. Figure S1(D-E)).

**Figure 1.**
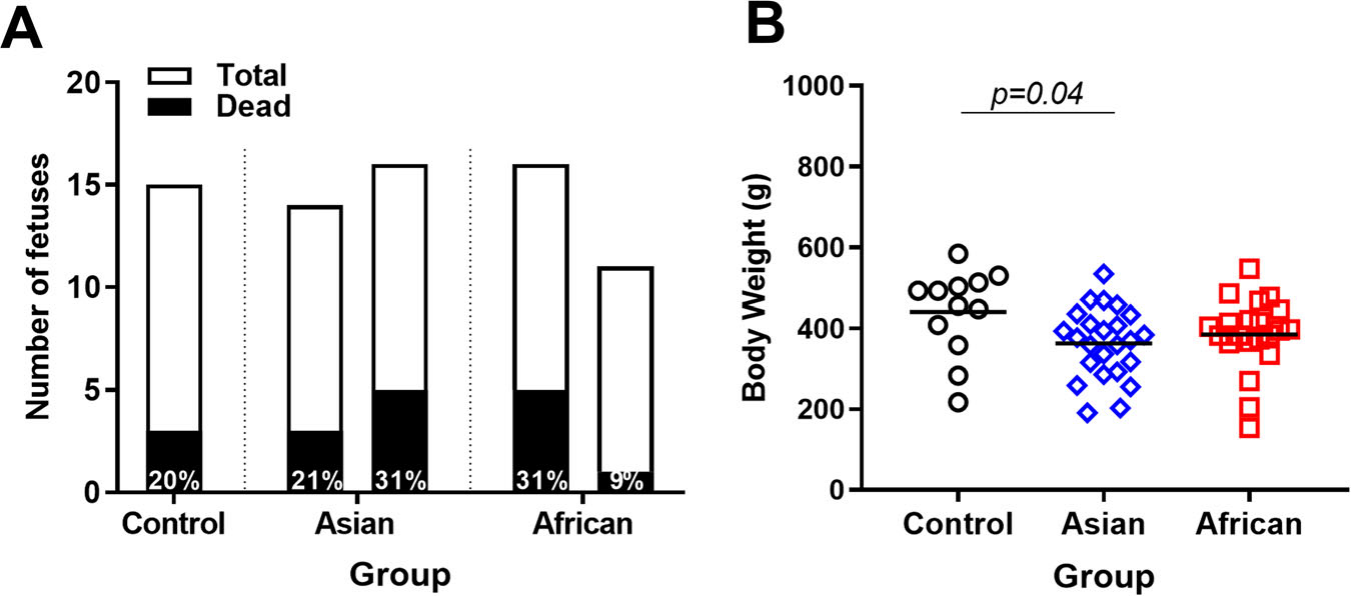
Fetal mortality and fetal body weights. (A) The total number of fetuses and the percentage of dead fetuses. (B) Body weights in control and ZIKV-exposed fetuses. Body weights of dead fetuses were not included. Solid lines represent mean values. Raw data are provided in Table S1(A).

A control litter contained three (20%) dead fetuses, which is in line with typical rates of fetal mortality in pigs of 7.5-30% [41, 42]. The number of dead fetuses was similar in African (9-31%) and Asian (21-31%) groups (*p* ≥ 0.82, Figure 1(A)). All dead fetuses from both African and Asian groups (except three fetuses from the Asian group) had high ZIKV loads in amniotic membranes (African: 6.3-8.7 log_10_ RNA copies/g; Asian: 5.2-7.8 log_10_ RNA copies/g). However, because the Control group had a similar number of dead fetuses, we do not know whether ZIKV infection was an actual cause of death in virus-exposed litters. Tissue decomposition also prevented virus testing in brains, placenta, and most body fluids from dead fetuses. Body weights of viable fetuses in the Asian group were significantly smaller than in the Control group but comparable between ZIKV experimental groups (Figure 1(B)).

**Figure 2.**
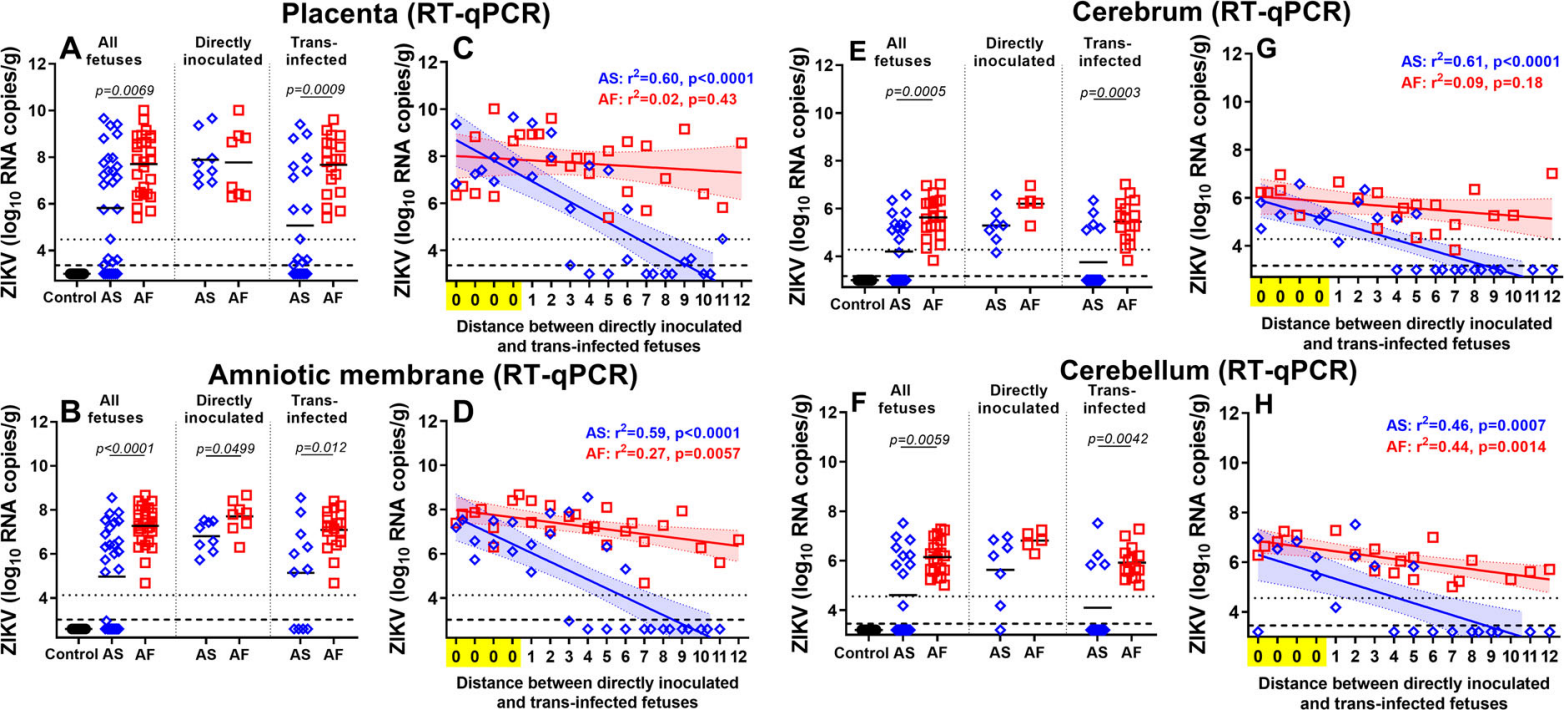
Kinetics of in utero ZIKV infection. ZIKV RNA loads in the placenta (A), amniotic membranes (B), fetal cerebrum (E), and cerebellum (F) of directly inoculated and trans-infected fetuses. Solid lines represent mean values. Linear regression shows the relationship between ZIKV loads in placenta (C), amniotic membranes (D), fetal cerebrum (G), and cerebellum (H) and the distance between directly inoculated and trans-infected siblings in the uterus. The x-axis for linear regression modeling: 0 – a directly inoculated index fetus; 1–12 is the distance from the directly virus-inoculated fetuses to a non-manipulated sibling. Blue and red lines represent the fitted regression lines with a shaded area representing the 95% confidence region for Asian and African groups, respectively. The dotted line represents the limit of quantification (LOQ). The dashed line represents the limit of detection (LOD). AS – the Asian group. AF – the African group. Raw data are provided in Table S1(B).

To compare the dynamics of ZIKV transmission between fetuses, we quantified viral loads in individual placental tissues and amniotic membranes from all directly inoculated and trans-infected fetuses. Virus titers in the placenta were significantly higher in fetuses inoculated with the African strain (Figure 2(A)). When we subdivided fetuses into directly inoculated, and trans-infected subgroups, directly inoculated fetuses did not show significantly different titers between African and Asian groups. In contrast, ZIKV titers in trans-infected fetuses were significantly higher in the African group (Figure 2(A)). Virus titers in amniotic membranes were significantly higher in all, directly inoculated, and trans-infected fetuses in the African group (Figure 2(B)). Zika virus RNA loads in the placenta and amniotic membrane of not manipulated fetuses from the Asian group strongly correlated with the distance to the directly inoculated index siblings (Figure 2(C-D)). This linear regression analysis suggests that ZIKV passes more efficiently from fetus to fetus in the African group than in the Asian group. In support, high ZIKV RNA loads were distributed more evenly in the placenta and amniotic membranes of non-manipulated fetuses in the African group than in the Asian group (Figure 2(A-D)). As in a previous study on porcine fetuses infected with the Asian ZIKV strain [35], here, we were not able to isolate the infectious virus from placental and amniotic tissues in both ZIKV groups. Other studies have also reported difficulties in isolating infectious ZIKV from human fetal tissues with pathology [43, 44] and tissues of immunocompetent animals [45]. Thus, to confirm productive infection, we tested all placental and amniotic samples in a PCR assay targeting the negative strand of ZIKV RNA [46] (Table S1(C)). In the African group the significantly higher number of samples (placenta - 89%; amnion - 85%) than in the Asian group (placenta - 50%; amnion - 50%) were positive for the negative strand of ZIKV RNA (placenta: *p* = 0.0041; amnion: *p* = 0.057) (Table S1(C)).

**Figure 3.**
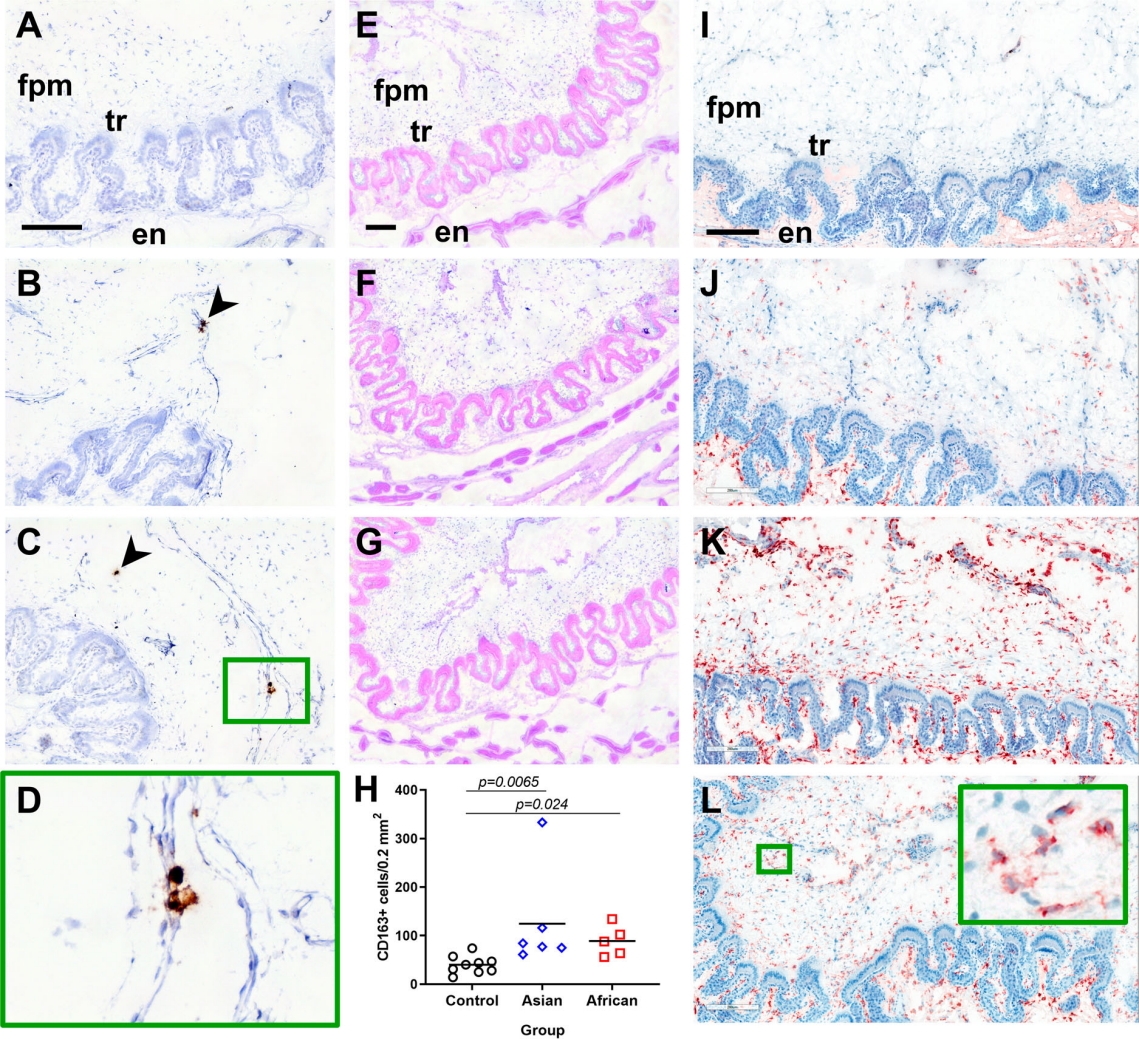
ZIKV infection in the placenta and placental responses. (A-D) ZIKV-specific ISH in placental samples. (A) Control samples from mock-exposed fetuses and tissues from ZIKV-exposed fetuses treated with ZIKV-specific and negative control probe, respectively, had no staining. Brown staining (arrowhead) indicates ZIKV positive-cells in fetal placental mesenchyme of Asian (B) and African (C) groups. ZIKV-positive cells were localized in fetal placental mesenchyme, while maternal endometrium and trophoblast were free for positive cells. Placental samples from four fetuses in each group were tested. ZIKV-positive cells were detected in one placental sample in both Asian and African groups. In the sample infected with the Asian strain, a single microscopic view contained virus-positive cells. In the sample infected with the African strain, multiple microscopic views contained virus-positive cells. (D) x400 magnification of ZIKV-positive cells detected by ISH. (E-G) Hematoxylin and eosin staining in the placenta. (H) Quantification of CD163-positive macrophages in fetal placental mesenchyme. In the Control group, in addition to placental samples collected in this study, we included control samples from two gestation-matched control pigs (G270 – 3 samples, and G312 – three samples [35]). CD163-specific staining (red) in the staining control (no primary Ab) (I), Control group (J), Asian group (K), and African group (L). The pattern of CD163 staining in the endometrium was the same between different groups. Insert in the green quadrat contains the x400 magnified field with positive cells. Identification of samples tested with ISH, hematoxylin and eosin, and stained for CD163 can be found in Table S1(B). fpm – fetal placental mesenchyme; tr – trophoblast; en – maternal endometrium. All scale bars are 200 μm.

Next, we localized ZIKV-positive cells in placental samples by ISH (Figure 3(A-D)). In both groups, ZIKV-positive cells were distributed within fetal placental mesenchyme. Trophoblast and maternal endometrium were negative for ZIKV.

We did not identify histopathology in fetal placenta mesenchyme (Figure 3(E-G)). However, both experimental groups showed increased numbers of CD163-positive cells in fetal placental mesenchyme (Figure 3(H-L)).

To further compare the dynamics of trans-fetal ZIKV transmission, we quantified viral loads in amniotic fluids from all fetuses. In high agreement with findings in placental and amniotic membrane tissues, ZIKV RNA loads in amniotic fluids were significantly higher in the African group (Suppl. Figure S2(A)). Next, we isolated and titrated infectious ZIKV from amniotic fluids which confirmed productive, persistent infection in both African and Asian groups (Suppl. Figure S2(B)). Infectious titers were not significantly different between groups; however, the trend to more intensive infection was apparent in the African group, particularly in amniotic fluids from trans-infected fetuses (Suppl. Figure S2(B)). Zika virus RNA loads in amniotic fluids of not manipulated fetuses from the Asian group correlated with the distance to the directly inoculated siblings (Suppl. Figure S2(C)). Similar to the linear regression analysis in the placenta and amniotic membranes, these data also suggest that ZIKV passes more efficiently from fetus to fetus in the African group. The similar trend, however, less pronounced, was detected when linear regression analysis was done on infectious virus titers in amniotic fluids (Suppl. Figure S2(D)).

Fetuses in the African group had considerably higher ZIKV loads in blood plasma (Suppl. Figure S2(E, G)) and ZIKV-specific Ab titers (Suppl. Figure S2(F, H)); however, the difference was not statistically significant. In the African group, 19% of fetuses showed negative-strand ZIKV RNA in blood plasma (Table S1(C)). In contrast, only 4% of fetuses in the Asian group showed negative ZIKV RNA in blood plasma (Table S1(C)).

### The African ZIKV strain causes higher viral loads in the fetal cerebrum and cerebellum than Asian strain

To compare infection phenotypes caused by African and Asian strains in fetal brains, we quantified viral loads in the cerebrum and cerebellum from directly inoculated and trans-infected fetuses. Viral loads in both cerebrum and cerebellum were higher in fetuses (all, directly inoculated, or trans-infected) exposed to the African strain (Figure 2(E, F)). Linear regression analysis of Zika virus RNA loads in fetal brains and the distance to the directly inoculated siblings was in a high agreement with results of regression analysis performed with placenta, amniotic membranes, and amniotic fluid samples, suggesting that ZIKV passes more efficiently from fetus to fetus in the African group than in the Asian group (Figure 2(G, H)). In support, while 100% of fetuses from the African group had ZIKV in the cerebrum and cerebellum, the considerable proportion of samples in the Asian group were negative (cerebrum: 50.0%; cerebellum: 52.4%; these negative samples were of fetuses which are most distant from directly inoculated siblings) (Figure 2(G-H)). Detection of negative-strand ZIKV RNA in the fetal cerebrum confirmed similar rates of productive persistent brain infection in both African and Asian groups (*p* = 0.89; Table S1(C)).

**Figure 4.**
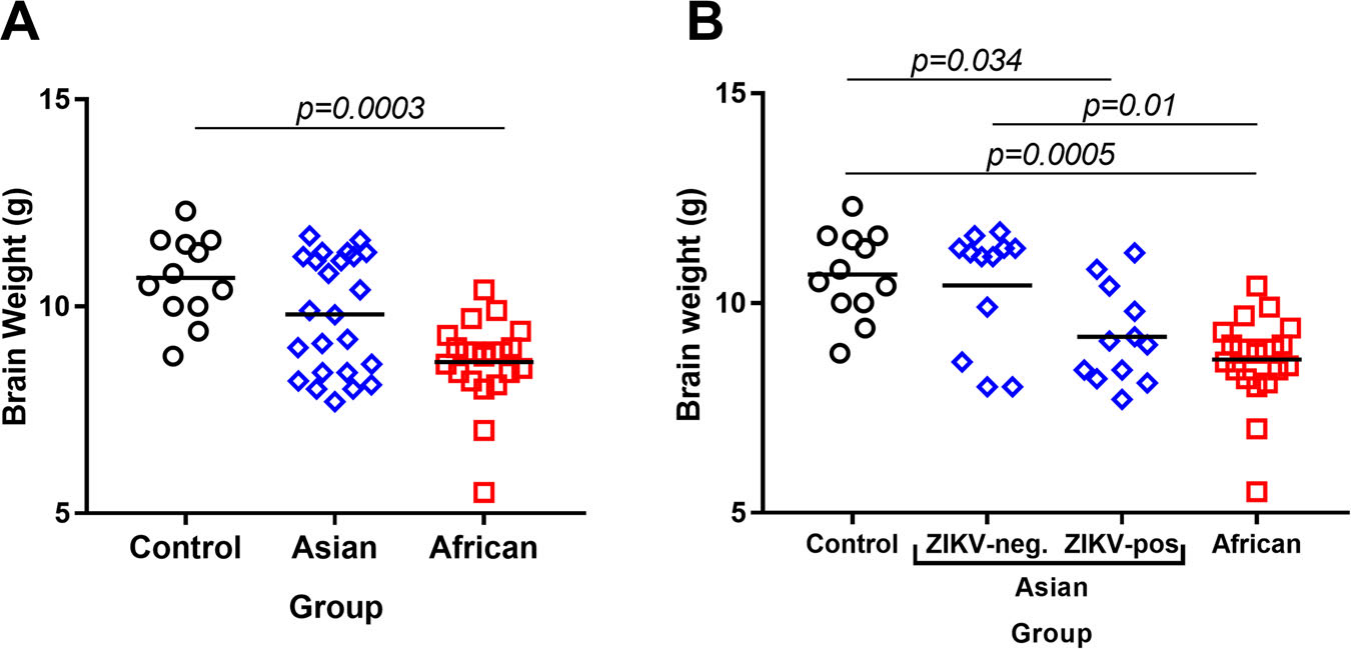
Fetal brain weights. (A) Fetal brain weights of all viable fetuses exposed to media (negative control), Asian ZIKV strain, or African ZIKV strain. (B) Fetal brain weights of all viable fetuses exposed to media, Asian ZIKV strain, or African ZIKV strain; the Asian group was subdivided into ZIKV-negative and ZIKV-positive subgroups based on the results of virus-specific RT-qPCR in the brain. Solid lines represent mean values. Raw data are provided in Table S1(A).

Brain weights in viable fetuses were significantly lower in the African group in comparison to the Control group (Figure 4(A)). The brain weights were also lower in the African group than in the Asian group; however, the difference was not statistically significant (*p* = 0.05). When we subdivided samples in the Asian group on ZIKV-negative and ZIKV-positive subgroups, virus-positive fetal brains had significantly lower weights than fetal brains in the Control group Figure 4(B). Similar to previous studies in pigs *in utero* infected with Asian strains at midgestation [35, 37], we did not find brain histopathology (hematoxylin and eosin staining) in viable fetuses in any group. The CD163 staining pattern in brains was similar in the Control and both ZIKV groups (Suppl. Figure S3).

### Infection caused by both Asian and African ZIKV strains is associated with elevated *in utero* IFN-α and cortisol levels

**Figure 5.**
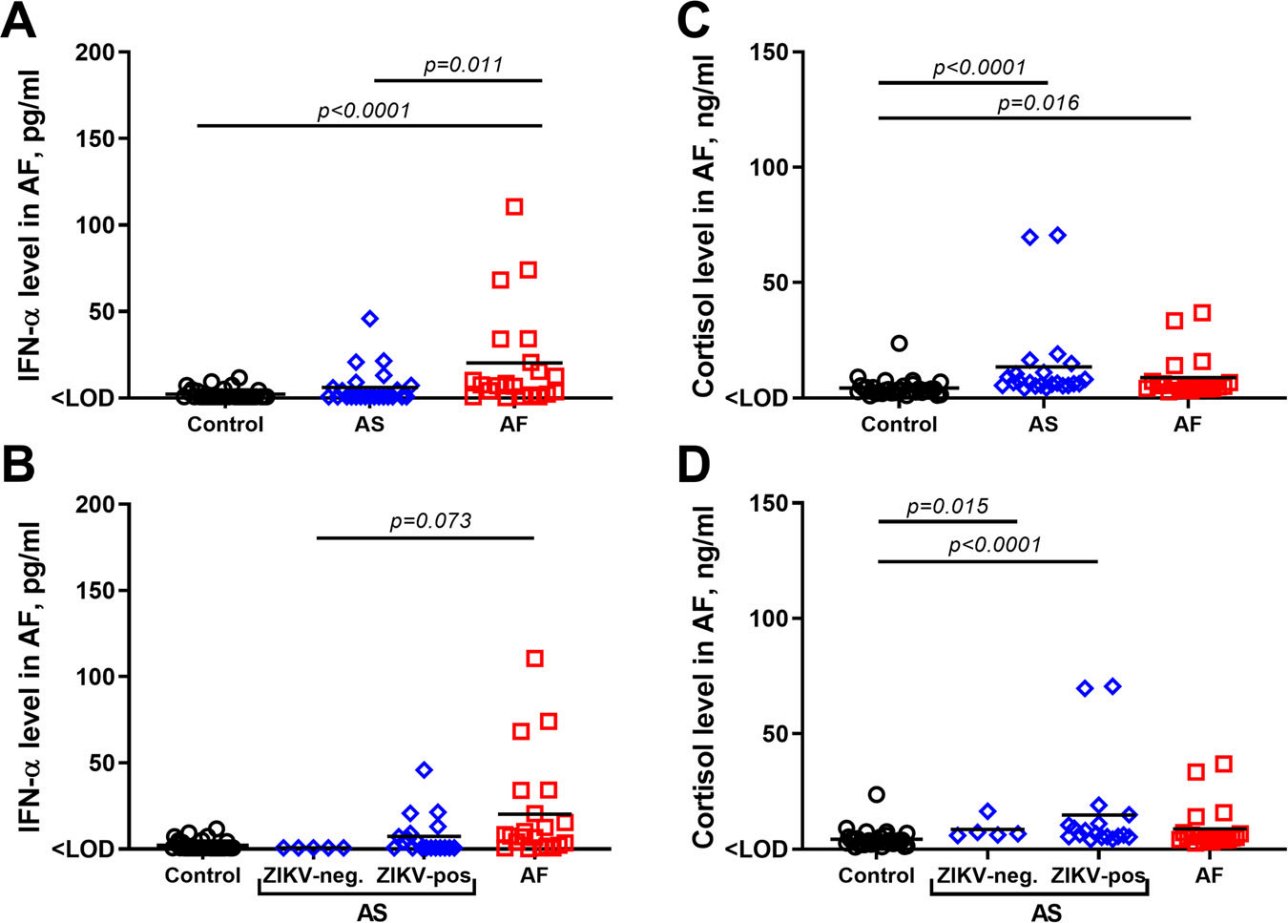
In utero IFN-α and cortisol levels. (A, B) IFN-α levels in amniotic fluid from Control, Asian (AS), and African (AF) groups. In the Control group, in addition to amniotic fluid samples collected in this study, we included previously published IFN-α data in samples from two gestation-matched control pigs (G270 and G312) [35]. (C, D) Cortisol levels in amniotic fluid from Asian and African groups. In the Control group, in addition to amniotic fluid samples collected in this study, we included previously published cortisol data in samples from two gestation-matched control pigs (G270 and G312) [36]. Solid lines represent mean values. In the Asian group, fetuses in the ZIKV-negative subgroup did not have detectable ZIKV RNA, infectious virus, or Ab in any tested samples. Raw data are provided in Table S1(E).

Persistent subclinical *in utero* infection with the Asian ZIKV strain in porcine mid-gestation increases IFN-α levels in amniotic fluid [35]. Here, the Asian group showed increased IFN-α levels in comparison to the Control group; however, the difference was not statistically significant (Figure 5(A)). Levels of IFN-α in the Asian group positively correlated with ZIKV titers in amniotic fluids (Spearman’s ρ = 0.68, *p* = 0.002). When we subdivided samples into the Asian group on ZIKV-negative and ZIKV-positive subgroups, increased IFN-α levels were exclusively attributed to virus-positive fetuses (Figure 5(B)). The African group showed significantly higher levels of IFN-α than Control group, Asian group, and Asian virus-negative subgroup (Figure 5(A, B)). The levels were not significantly different between the African group and Asian virus-positive subgroup (*p* = 0.20). Levels of IFN-α in the African group did not correlate with ZIKV titers in amniotic fluids (*p* = 0.92), most probably because amniotic fluids from all fetuses in the African group had consistently high and uniform ZIKV loads (Suppl. Figure S2(A-D)).

Amniotic fluids are also used to assess prenatal cortisol exposure in humans and associated developmental sequelae in offspring [47]. In our previous study, we demonstrated that persistent infection in porcine conceptuses caused by the Asian ZIKV strain was associated with increased cortisol levels in amniotic fluids [36]. In this study, *in utero* cortisol levels in African and Asian groups, and Asian subgroups were also significantly higher than in the Control groups (Figure 5(C-D)).

## Discussion

This is the first non-rodent, large animal model study, which demonstrated that the African strain of ZIKV, with no passage history in experimental animals, can cause persistent *in utero* infection at midgestation. We showed that the African strain caused more vigorous *in utero* infection as indicated by a more efficient virus spread from fetus to fetus, higher viral loads in the placenta, amniotic membranes, amniotic fluids, and fetal brains. Four fetuses were directly inoculated in each pig with the African or Asian ZIKV strain (Suppl. Figure S1(B)). After inoculation, the virus initially spreads from the index conceptuses to non-manipulated adjacent, and subsequently to more distant siblings providing an *in utero* transmission assay to compare the dynamics of trans-fetal infection between African and Asian strains. Each porcine conceptus has its amniotic membrane and placenta (Suppl. Figure S1(D)). At the mid of gestation, adjacent pig fetuses located within the uterus have physical contact between fetal placental membranes (allantochorion) [48], and cell trafficking between porcine fetuses has been previously demonstrated [49]. Most probably in our model, the virus spreads from fetus to fetus via adjacent fetal placental membranes (Suppl. Figure S1(C-D)). One of two mothers in both African (9.3 log_2_) and Asian (7.0 log_2_) groups had ZIKV-specific Ab suggesting the virus spread from uteri to maternal tissues and transient productive infection (Table S1(D)). We did not detect ZIKV RNA in the maternal blood plasma (RT-qPCR at 28 days after inoculation) and endometrium (ISH). Also, in our previous studies, maternal blood, endometrium, and lymph nodes were free of ZIKV [35, 36]. Moreover, direct intravenous maternal inoculation with a high ZIKV dose (10^6.4^ TCID_50_) did not result in fetal infection [37]. Altogether, the ZIKV spread from mother to fetus is unlikely; however, additional studies are required to prove this.

Our data suggest that the African strain may have a stronger tropism to the porcine placenta and amniotic membranes than the Asian strain as indicated by higher viral loads. These findings are in agreement with previous *in vitro* studies where African ZIKV strains replicated more efficiently in human placental cells than Asian strains [50]. In our previous fetal pig model study [35] and here, virus-specific *in situ* hybridization showed that ZIKV replication is restricted to fetal placental mesenchyme. The same virus tropism has been described in the human placenta [51]. Fetal placental mesenchyme (allantochorion) in pigs has all major cell types found in the human fetal side of the placenta (chorionic plate) and performs the same fundamental functions [52–54] Thus, while different placentation types in pigs (epitheliochorial) and humans (hemochorial) prevent modeling mechanisms of virus transmission from mother to fetus, the porcine model is a useful tool for comparing infection kinetics of different ZIKV strains in fetal membranes and may be used to study strain-specific virulence factors *in vivo*. It is particularly important because ZIKV heterogeneity in the Americas has been established [1].

*In utero* infection with the African strain was characterized by higher viral loads in fetal brains. The African strain caused infection in all tested fetal brains that was associated with significantly lower brain weights in comparison to the Control group. In contrast, the Asian group did not show a significant difference in fetal brain weights (Figure 4). However, the Asian strain was detected only in 50.0% of tested brains. Following analysis of samples subdivided on ZIKV-negative and ZIKV-positive subgroups within the Asian group demonstrated that virus-positive brains were significantly smaller than brains in the Control group (Figure 4(B)). Microencephaly, a phenotype with normal head dimensions and abnormally small brain size, has been described in pig fetuses infected with the Asian ZIKV strain [37]. Altogether, our data suggest that both Asian and African strains of ZIKV could lead to microencephaly in porcine fetuses. Pathogenesis of microencephaly is currently unknown. In addition to direct effects of virus infection in the brain, replicating in the placenta for around a month, ZIKV may affect the developing fetal brain distantly contributing to observed microencephaly. Similar to previous studies in the porcine model [37] and humans [51], here, placental samples did not have histopathological lesions like necrosis or hemorrhages. However, the increased number of CD163-positive cells was observed in placental samples in both Asian and African ZIKV groups. The increased number of CD163-positive cells has been also described in the ZIKV-infected human placenta [51]. The hemoglobin scavenger receptor, CD163, is a macrophage-specific marker and its upregulation was associated with placental dysfunction in preeclampsia and stillbirth [55, 56]. Further studies are warranted to discriminate the effects of local ZIKV infection in the fetal brain and distant infection in the placenta.

We have previously demonstrated that persistent subclinical infection with the Asian ZIKV strain in porcine mid-gestation increases *in utero* levels of IFN-α [35]. Here, fetuses infected with Asian or African ZIKV strains showed elevated *in utero* IFN-α levels. Congenital ZIKV infection in mice also increased *in utero* levels of type I IFNs [34, 57], which was suggested to play a role in fetal pathology [57]. Thus, it is important to study the pathological role of altered IFN-α responses during *in utero* infection caused by both Asian and African strains.

In addition to cytokines, *in utero* hormonal balance is important to fetal and offspring health [58]. In our previous study, we demonstrated that persistent infection in porcine conceptuses caused by the Asian strain was associated with increased *in utero* cortisol levels [36]. In this study, infection with both Asian and African strains was repeatedly associated with increased *in utero* cortisol levels. The data suggest that different lineages of ZIKV can lead to *in utero* cortisol pathology.

The recent epidemic in the Americas instigated heightened attention to ZIKV and microcephaly worldwide. As a result, the absence of congenital Zika syndrome in Africa has been challenged, suggesting that the Zika epidemic in Africa could be overlooked [5–11, 18, 19]. In support, previous studies in pregnant mice [16, 17] and present work in the fetal pig model systematically demonstrate that African strain of ZIKV can cause more vigorous *in utero* infection and pathology. Interestingly, pathology in viable fetuses was not apparent until brains were weighted, placental samples were stained for the specific cell marker, and *in utero* cortisol and IFN-α levels were quantified. Thus, similarly to Asian strains [3, 20, 21, 59], ZIKV of African lineage might cause silent pathology that is difficult to identify in asymptomatic fetuses and offspring. Altogether, these findings emphasize the need for further studies to highlight the impact of ZIKV heterogeneity on infection outcomes during pregnancy. Systemic research to comprehend ZIKV epidemiology in Africa and estimate potential risks in the case of the introduction of African strains to new territories with different population immunity is highly needed.

## Supplementary Material

Supplemental Material
