# Supplementary material for: The African strain of Zika virus causes more severe in utero infection than Asian strain in a porcine fetal transmission model
Source: Emerg Microbes Infect. 2019 Jul 25;8(1):1098–107. doi: 10.1080/22221751.2019.1644967 (PMC6711198; doi:10.1080/22221751.2019.1644967)
Supplement: Supplemental Material [file TEMI_A_1644967_SM8325.zip › Supplemental Material and Methods_fnal.docx]

**The African strain of Zika virus causes more severe *in utero* infection than Asian strain in a porcine fetal transmission model**

Daniel Udenze^1,2†^, Ivan Trus^1†^, Nathalie Berube^1^, Volker Gerdts^1,3^, Uladzimir Karniychuk^1,2,3*^

^1^Vaccine and Infectious Disease Organization-International Vaccine Centre (VIDO-InterVac), University of Saskatchewan, Saskatoon, SK S7N 5E3, Canada

^2^School of Public Health, University of Saskatchewan, Saskatoon, SK S7N 2Z4, Canada

^3^Department of Veterinary Microbiology, Western College of Veterinary Medicine, University of Saskatchewan, Saskatoon, SK S7N 5B4, Canada

†These authors contributed equally to this work.

***Corresponding author**: Vaccine and Infectious Disease Organization, International Vaccine Centre (VIDO-InterVac), University of Saskatchewan, Saskatoon, SK S7N 5E3, Canada. Email: u.karniychuk@usask.ca

Email addresses:

DU: dou286@mail.usask.ca

IT: ivan.trus@usask.ca

NB: nab602@mail.usask.ca

VG: volker.gerdts@usask.ca

UK: u.karniychuk@usask.ca

**Supplemental Materials and Methods**

**Virus**

We used the low-passage, contemporary (Puerto Rico, 2015), Asian ZIKV strain PRVABC59 [GenBank: KU501215.1] isolated from human serum specimen and historical (Senegal, 1984) African ZIKV strain DAK-AR-41524 (BEI resources) [Genbank: KY348860.1]. These strains were never passaged in experimental animals. After two passages on C6/36 cells, cell culture media containing ZIKV was centrifuged (12 000g, 20 min, +4°C), and the supernatant was collected. Media from virus-negative C6/36 cells was used for mock-inoculation. Viral titers were quantified with endpoint dilution assay as described below. The absence of mycoplasma contamination in all virus stocks and cell cultures was confirmed using LookOut Mycoplasma PCR Detection Kit (Sigma-Aldrich).

**Animal experiments**

Animal experiments were performed in strict accordance with the Canadian Council on Animal Care guidelines for humane animal use. All animal protocols were approved by the University of Saskatchewan's Animal Research Ethics Board. Five, pregnancy-matched Landrace-cross pigs, were obtained from a high-health status herd free for porcine reproductive and respiratory syndrome virus (PRRSV) and porcine parvovirus (PPV) (viruses which can cause fetal infection in pigs). Selected fetal samples were tested by PCR for PRRSV and PPV^1^. Also, fetal samples were tested for porcine circovirus 2 (another pig virus which can cause infection in fetuses) using susceptible cells and home-made PCR assay. Tested samples were negative for all tested pathogens. Pregnant pigs were housed at the Vaccine and Infectious Disease Organization-International Vaccine Centre (VIDO-InterVac) biosafety level 2 containment facility. Pregnant pigs were randomly assigned into control (one animal), African and Asian groups (two animals per group). All animals were housed in identical, but isolated rooms. Housing conditions and diet were the same for all sows.

*In utero* inoculation was performed at 50 gestation days (gd) (the total duration of porcine pregnancy is 114–115 days) as previously described^2^. For precise inoculation, we used an ultrasound-guided technique which allows verifying fetal viability before and after injection by visualizing heart beating. We inoculated four conceptuses (a fetus with fetal membranes) per pig. Animals in corresponding groups were inoculated with 10^6^ TCID_50_/conceptus of African (pigs #553-5E and #482-3E) or Asian (pigs #553-1E and #553-2E) ZIKV strains intraperitoneally + intra-amniotic (IP+IA; 100 μl+100 μl) (**Table S1(A)**). Four conceptuses from the control pig (pig #516-4E) were inoculated with virus-free media.

Pregnant pigs were euthanized and sampled 28 days post in utero inoculation (dpi). Uteri with fetuses were removed. Amniotic fluid, amniotic membranes, and uterine wall with the placenta (fetal placental compartment was subsequently dissected from the maternal endometrium) were collected from each conceptus and rapidly frozen. Amniotic fluids were aspirated with sterile syringes with needles before tissue dissection. All samplings were performed in the direction from a non-manipulated conceptus closest to the right uterine horn tip toward inoculated fetuses. Fetuses were visually examined. Blood plasma was collected from all fetuses. Fetal brains were removed and dissected away in the same way from all fetuses. Body and brain weights of viable fetuses were measured. The fetal brain (cerebrum with cerebellum) was frozen in liquid nitrogen. A part of the frontal brain lobe and uterine wall with the placenta from selected fetuses were dissected and fixed in formalin for subsequent hematoxylin and eosin staining to screen lesions. We also collected blood samples from mothers at inoculation and euthanasia. After blood centrifugation (2000g, 20 min, +4°C), plasma was aliquoted and immediately frozen (-80°C).

**RNA extraction and RT-qPCR**

PureLink RNA Mini Kit (Invitrogen) was used to extract RNA from amniotic fluid and blood plasma according to the manufacturer’s instructions. RNA from amniotic membranes, placenta, cerebrum, and cerebellum was extracted using QIAamp Viral RNA Mini Kit (QIAGEN, USA) as previously described^1^. Before RNA extraction, tissue samples were weighed on an analytical balance. First, tubes with homogenization beads were preweighed. Subsequently, tube + bead weight was subtracted from tube + bead + tissue weight. Tissues were homogenized in 560 μl AVL lysis buffer (amniotic membranes, placenta) or 1 ml QIAzol Lysis Reagent (fetal cerebrum and cerebellum) using RNase-free stainless steel beads and TissueLyser II (QIAGEN, USA) operating for 5 min at 25 Hz.

Previously described probe-based one-step real-time reverse transcriptase-polymerase chain reaction (RT-qPCR) assay with modifications was used for ZIKV RNA quantification^3^. All RT-qPCR reactions were conducted on the StepOne Plus platform (Life Technologies, USA) and analyzed using StepOne software version 2.3. RT-qPCR reaction mixture (ref.: Bioline BIO-77005) (20 μl) consisted of 10 μl 2x SensiFAST Probe One-Step Mix, 0.4 μl RiboSafe RNase Inhibitor, 0.2 μl reverse transcriptase, 1 μl (500 nM) of forward and reverse primers, 0.5 μl (250 nM) probe (ZP-N: 5′-FAM-AGCCTACCT-ZEN-TGACAAGCAATCAGACACTCAA-IABkFQ-3′), 1.9 μl nuclease-free water and 4 μl RNA. To obtain the highest sensitivity, different primer sets were used to quantify the Asian (ZIKV-AS-F1086: 5′-CCGCTGCCCAACACAAG-3′ and ZIKV-AS-R1126: 5′-CCACTAACGTTCTTTTGCAGACAT-3′) and African (ZIKV-AF-F1086: 5′-CCGTTGTCCAACACAAG-3′ and ZIKV-AF-R1126: 5′-CCACCAATGTTCTCTTGCAGACAT-3′) strains. A reverse transcription step of 10 min at 48 °C and an enzyme activation step of 2 min at 95 °C were followed by 60 amplification cycles (10 s at 95 °C and 20 s at 60 °C). RNA from a stock of PRVABC59 and DAK-AR-41524 ZIKV strains was used two generate strain-specific standard curves. Both standard curves had a wide dynamic range (10^2.1^-10^7.7^ copies/reaction) with the high linear correlation (PRVABC59: R^2^ = 0.9987, DAK-AR-41524: R^2^ = 0.9953) between the cycle threshold (Ct) value and template concentration. The standard curve was used to determine detection (Ct 40) and quantification (Ct 36) limits as previously described^4^. PCR values were corrected for fluid volumes or tissue weights and upon logarithmical transformation expressed as ZIKV RNA genome copies per ml or gram.

Productive ZIKV infection in brain tissues (cerebrum), placenta, amniotic membrane, and fetal blood plasma was confirmed with ZIKV negative strand RT-PCR^5^. cDNA was synthesized with SuperScript III First-Strand Synthesis System (Invitrogen) using 10 pmole of the ZIKV-835 forward primer (PRVABC59: 5’-TTGGTCATGATACTGCTGATTGC-3’, DAK-AR-41524: 5’-TTGGTCATGATATTGTTGATTGC-3’). Afterward, cDNA was amplified using the primers (ZIKV-AS-F1086 and ZIKV-AS-R1126 for the PRVABC59 strain, ZIKV-AF-F1086 and ZIKV-AF-R1126 for the DAK-AR-41524 strain; 500 nM of each) and probe (ZP-N; 250 nM) described above. An enzyme activation step of 5 min at 95 °C was followed by 45 amplification cycles (10 s at 95 °C and 20 s at 60 °C).

In all PCR assays, we used Vero E6 cell culture media containing ZIKV as a positive PCR control. As a negative control, we used samples from mock-inoculated and non-manipulated control fetuses. Strict precautions were taken to prevent PCR contamination. Aerosol-resistant filter pipette tips and disposable gloves were always used. Kit reagent controls were included in every RNA extraction and PCR run.

**Virus titration in amniotic fluid**

Amniotic ﬂuids were serially diluted fourfold in four replicates starting from undiluted, and 50 μl of each dilution was added to conﬂuent Vero E6 cells cultured in 96-well plates. Dilutions were made in DMEM media (Thermo Fisher Scientiﬁc, USA) supplemented with 1% fetal bovine serum (FBS) (Sigma-Aldrich, USA), a mixture of antibiotics (1000 IU/ml penicillin and 1 mg/ml streptomycin, Gibco) and 2.25 g/l Sodium Bicarbonate. After 2 h of incubation, 150 μl of fresh media was added to each well. The cells were incubated for 7 days. After plate washing and drying, the plates were kept at -20 °C at least for 2 h or until use. Fixation and staining were done, as previously described^2^. The anti-pan flavivirus E protein monoclonal antibodies (Ab) 4G2 (ATCC, HB-112) were used to detect ZIKV-infected cells. Fifty percent endpoint titers were calculated by the Spearman-Kärber formula and expressed in a decimal logarithm of a 50% infection dose for cell cultures (log_10_ TCID_50_). As a positive control, we used Vero E6 cell culture media containing ZIKV. Amniotic ﬂuid from mock-inoculated and non-manipulated control piglets were used as negative controls.

**ZIKV isolation from tissues**

Cell culture-based procedure for isolating the infectious ZIKV on C6/36 cells was previously described^6,7^. Selected placental and amniotic membrane (two samples from each pig) homogenates were inoculated directly into 6-well plates containing C6/36 cells. After 24 h at 28°C (in a parallel experiment, cells were incubated at 37°C for the first 24 h), supernatants were removed and replaced with media. The cells were cultured at 28°C in 5% CO_2_ for seven days. On day 7, the culture supernatant was collected and passaged on naïve C6/36 cells. Serial passages were conducted three times. The viral RNA was extracted from culture supernatants at each passage, and RT-PCR was performed as described above. The increase in RT-qPCR Ct values indicates the presence of infectious ZIKV. As a positive control, we used amniotic fluid containing ZIKV. Tissue homogenates from mock-inoculated fetuses were used as negative controls.

**Bio-Plex assay**

An assay in 96 well Grenier Bio-One Fluotrac 200 96F black plates (VWR, USA) was performed as previously described^1^. Bio-Plex assay reagents are listed in the table below.

**Bio-Plex assay reagents**

| **Cytokine** | **Capture antibody** | **Detection antibody** | **Standard** | **Bead** |
| --- | --- | --- | --- | --- |
| **IFN-α** | MAb anti porcine IFN-α; GeneTex GTX11408 | MAb anti-pig IFN-α; PBL 27105-1; biotinylated in house; 1:5000 dilution | Recombinant porcine IFN-α; Genentech; 200 pg/mL | Region 45; BioRad MC10045-01 |

**ZIKV-specific *in situ* hybridization**

RNA in situ hybridization (ISH) was performed with RNAscope 2.5 HD Reagent Kit-BROWN (Advanced Cell [Diagnostics](https://www.sciencedirect.com/topics/medicine-and-dentistry/diagnosis), USA) according to the manufacturer's instructions for frozen tissues^1^. The RNAscope V-ZIKV probe targeting ZIKV RNA (catalog no. 467771), positive control Sc-PPIB probe targeting pig [PPIB](https://www.sciencedirect.com/topics/biochemistry-genetics-and-molecular-biology/ppib) [gene](https://www.sciencedirect.com/topics/biochemistry-genetics-and-molecular-biology/nested-gene) (catalog no. 428591), and RNAscope negative control DapB probe (catalog no. 310043) probes were designed and synthesized by Advanced Cell Diagnostics. Tissues were counterstained with hematoxylin and visualized with standard bright-field [microscopy](https://www.sciencedirect.com/topics/biochemistry-genetics-and-molecular-biology/microscopy). To monitor the specificity of the staining, tissues from mock-exposed and ZIKV-exposed fetuses were treated with ZIKV-specific and negative control probes, respectively.

**CD163 staining**

Staining was performed as previously described^8^ with some modifications. Briefly, placental cryosections of 12 μm were fixed in methanol at -20°C for 15 minutes. After treatment with 0.3% H_2_O_2_ (10 min, RT), tissue sections were incubated with 2A10/11 [IgG_1_](https://www.sciencedirect.com/topics/immunology-and-microbiology/immunoglobulin-g1) mouse monoclonal antibodies (mAb; dilution 1:100) (Bio-Rad) against porcine [CD163](https://www.sciencedirect.com/topics/immunology-and-microbiology/cd163) for 1h at 37°C. Afterward, the sections were incubated with EnVision+ Single Reagents (HRP, mouse, Agilent) following Lab Vision™ Ready-To-Use AEC Substrate System (Thermo Fisher Scientific) according to the manufacturer’s instructions. Subsequently, tissues were counterstained with hematoxylin. The entire tissue sections were scanned using Aperio ScanScope XT (Leica Biosystems) at a magnification of x200, and images were analyzed using Image Scope Rev v12.1.0.5029 software. We quantified CD163-positive cells in fetal placental mesenchyme because ZIKV-positive cells were identified in the fetal part of the placenta. Five fields of fetal placental mesenchyme covering 0.2 mm^2^ were selected blindly for the quantification of CD163-positive cells.

**Serology**

For quantification of ZIKV-specific IgG Ab, immunoperoxidase monolayer assay (IPMA) was used as described before^1,2^. Briefly, Vero E6 cells in 96-well cell culture plates were inoculated with 50 μl media containing 5 TCID_50_ of the ZIKV PRVABC59 strain and incubated (2h, +37 °C, 5% CO_2_). Then 100 μl of the culture medium (DMEM + 5% FCS + 1% Penicillin/Streptomycin + 0.2% Sodium Bicarbonate) was added and after incubation (72h, +37°C, 5% CO_2_) plates were dried and stored at -20°C until use. Plates were thawed and cells were fixed in 10% buffered formalin (30 min, RT). Cells were washed twice with 1x PBS (pH 7.2) and incubated with 100% methanol in the presence of 0.3% H_2_O_2_ (10 min, RT). Then plates were washed with PBS and two-fold [serial dilutions](https://www.sciencedirect.com/topics/medicine-and-dentistry/serial-dilution) of blood plasma were added, followed by incubation (1h, +37 °C). Plates were washed three times with PBS containing 0.05% Tween 80 and 50 μl/well of rabbit anti-pig IgG conjugated with horseradish peroxidase (1:400 dilution, ab 136735, Abcam, USA) was added. After incubation (1h, +37 °C) and washing, color reaction was initiated by adding substrate solution (1 mM 3-amino-9-ethylcarbazole, 5% N,N-dimethylformamide, 50 mM Sodium Acetate (pH 5.0), 0.023% H_2_O_2_). The reaction was stopped by replacing the substrate with an acetate buffer and ZIKV-specific staining was determined by examination with a microscope. The titers were defined as the reciprocal logarithm of 2 of the highest serum dilution. Blood plasma from mock-inoculated and non-manipulated control fetuses and gilts were used as negative controls.

**Cortisol ELISA**

To profile *in utero* cortisol levels in amniotic fluids, we used ELISA Kit (ADI-900-071, Enzo Life Sciences, Farmingdale, NY, USA) according to the manufacturer’s instructions. The kit has been validated by the manufacturer for compatibility with porcine samples and has been previously used in pig research^2^. The assay detection limit was 56.7 pg/ml. The intra-assay coefficients of variability (CVs) were 10.5% (low), 6.6% (medium) and 7.3% (high). The inter-assay CVs were 13.4% (low), 7.8% (medium) and 8.6% (high).

**Supplemental References**

1 Darbellay J, Cox B, Lai K *et al.* Zika Virus Causes Persistent Infection in Porcine Conceptuses and may Impair Health in Offspring. *EBioMedicine* 2017. doi:10.1016/j.ebiom.2017.09.021.

2 Trus I, Darbellay J, Huang Y *et al.* Persistent Zika virus infection in porcine conceptuses is associated with elevated *in utero* cortisol levels. *Virulence* 2018; : 21505594.2018.1504558.

3 Faye O, Faye O, Dupressoir A, Weidmann M, Ndiaye M, Alpha Sall A. One-step RT-PCR for detection of Zika virus. *J Clin Virol* 2008; **43**: 96–101.

4 Forootan A, Sjöback R, Björkman J, Sjögreen B, Linz L, Kubista M. Methods to determine limit of detection and limit of quantification in quantitative real-time PCR (qPCR). *Biomol Detect Quantif* 2017; **12**: 1–6.

5 Nem de Oliveira Souza I, Frost PS, França J V *et al.* Acute and chronic neurological consequences of early-life Zika virus infection in mice. *Sci Transl Med* 2018; **10**: eaar2749.

6 Deng C, Liu S, Zhang Q *et al.* Isolation and characterization of Zika virus imported to China using C6/36 mosquito cells. *Virol Sin* 2016; **31**: 176–9.

7 Darbellay J, Lai K, Babiuk S *et al.* Neonatal pigs are susceptible to experimental Zika virus infection. *Emerg Microbes Infect* 2017; **6**. doi:10.1038/emi.2016.133.

8 Karniychuk UU, Nauwynck HJ. Quantitative Changes of Sialoadhesin and CD163 Positive Macrophages in the Implantation Sites and Organs of Porcine Embryos/Fetuses During Gestation. *Placenta* 2009; **30**. doi:10.1016/j.placenta.2009.03.016.
